# Prevalence and correlates of mental disorders among Chinese overseas students during the COVID-19: A multi-regional cross-sectional analysis

**DOI:** 10.1371/journal.pone.0303283

**Published:** 2024-05-13

**Authors:** Yijia Gao, Yuanyan Ma, Yaxin Li, Yuanji Zhao, Zhen Zeng, Xiaozhi Yao, Yingjun Nie

**Affiliations:** 1 College of the Arts, Wuhan Sports University, Wuhan, China; 2 College of the Sports, Huazhong Normal University, Wuhan, China; 3 School of Economics & Management, Wuhan Sports University, Wuhan, China; 4 College of the Physical Education, Wuhan Sports University, Wuhan, China; 5 Editorial Department, Wuhan Sports University, Wuhan, China; Kwame Nkrumah University of Science and Technology, GHANA

## Abstract

**Background:**

The global impact of the COVID-19 pandemic extends beyond physical health, significantly affecting mental health. Chinese overseas students are particularly susceptible to the adverse psychological effects of the pandemic. Understanding the prevalence and correlates of mental disorders in this population is essential for developing targeted interventions and support systems.

**Methods:**

Employing a snowball sampling technique, this study recruited Chinese overseas students from diverse regions. The 50-item Self-evaluation Table was utilized to assess the presence of mental disorders. Descriptive statistics, including percentages, 95% confidence intervals, means, and standard deviations, characterized the survey population. The chi-square test identified disparities among categorical variables, while logistic regression explored risk factors for mental disorders among Chinese overseas students.

**Results:**

Out of the total sample size of 10,864 Chinese overseas students, a staggering 7,090 (65.4%) met the diagnostic criteria for mental disorders. Furthermore, the degree of mental disorder varied significantly across different regions (p < 0.001), education levels (p < 0.05), the duration of anti-epidemic measures (p < 0.05), and age (p < 0.05), while no significant differences were observed in terms of gender (p > 0.05). Several risk factors contributing to the mental disorder burden among Chinese overseas students during the pandemic were identified, including the seriousness of the epidemic in their residential area, the apprehension of getting infected, anxieties regarding academic performance, the infection control policies implemented by the host government, preventive measures taken locally to counter the epidemic, and challenges encountered in returning to their home country.

**Conclusion:**

Given the significant challenges in mental health faced by Chinese overseas students during the COVID-19 crisis, addressing their specific needs and implementing tailored measures is imperative. Future public health emergencies should consider the potential mental disorders and disease risks faced by Chinese overseas students. By providing comprehensive support and targeted interventions, policymakers, educational institutions, and healthcare providers can help mitigate the adverse psychological effects and promote the well-being of this vulnerable population.

## Introduction

The COVID-19 pandemic, declared a Public Health Emergency of International Concern (PHEIC), poses significant challenges to the implementation of effective global control measures. Educational institutions worldwide have swiftly enacted measures to minimize the risk of student infection and ensure continuous learning, including temporary school closures, class rescheduling, virtual learning transitions, and exam postponements. UNESCO data indicates that nearly 300 million international higher education students worldwide have already been affected by the spread of the pandemic (UNESCO 2020). This unprecedented crisis has impacted the physical and mental well-being of international students, leading to notable alterations in sleep patterns, dietary habits, physical activity, and overall quality of life [1, 2]. Despite global policy implementations, effectively managing the pandemic remains challenging, and individuals lack effective treatment strategies beyond social distancing and home isolation [3], resulting in adverse psychological consequences.

In an increasingly globalized world, the number of Chinese students studying abroad has surged to 15.3 million, constituting one-fourth of all international students globally(CCG 2022). These students often face distress due to negative experiences such as anti-Chinese sentiment and racial discrimination [4], rendering them more susceptible to mental disorders like anxiety, depression, and feelings of betrayal [5]. Some even experience isolation in certain countries, being labelled as potential SARS-CoV-2 carriers [6]. A study by Song (2020) during the pandemic found that over 30% of surveyed Chinese overseas students rated their mental health as moderate to severe, with nearly 50% reporting similar levels of anxiety [4]. Concurrent research in North America highlighted that around half of Chinese international students grappled with significant psychological pressures [7].

Previous research has illuminated the profound impact of the pandemic on the psychological well-being of college students, including those studying abroad. Indicators such as somatization, anxiety, depression, stress, and fear are commonly used to assess mental health. A meta-analysis of data from 47 countries revealed a significant increase in the global prevalence of depression, anxiety, and stress during the COVID-19 pandemic. Yet, amidst this crisis, the precise influence of various factors on the prevalence of mental disorders among Chinese overseas students remains uncertain. While prior studies have addressed the severe psychological toll on international students during epidemics, a notable research gap persists regarding the assessment of the impact of psychological barriers, their underlying causes, and potential regional disparities that may exacerbate their effects. A precise understanding of these influential factors is crucial for implementing targeted interventions to address the mental health challenges faced by Chinese overseas students.

In the foreseeable future, the potential impact of large-scale public health events on the educational pursuits and overall mental health of Chinese overseas students cannot be ignored. This study’s primary objective is to illuminate the prevalence and correlations of mental disorders among Chinese overseas students during the outbreak through a large-scale survey with a substantial sample size. Systematic data collection and analysis will provide a comprehensive understanding of the prevailing psychological state within their environment, forming the basis for scientifically rigorous and practical intervention programs. These programs aim to identify and address potential issues early on, thereby alleviating the mental and physical burdens faced by Chinese overseas students.

## Methods

### Participants

**Fig 1:**
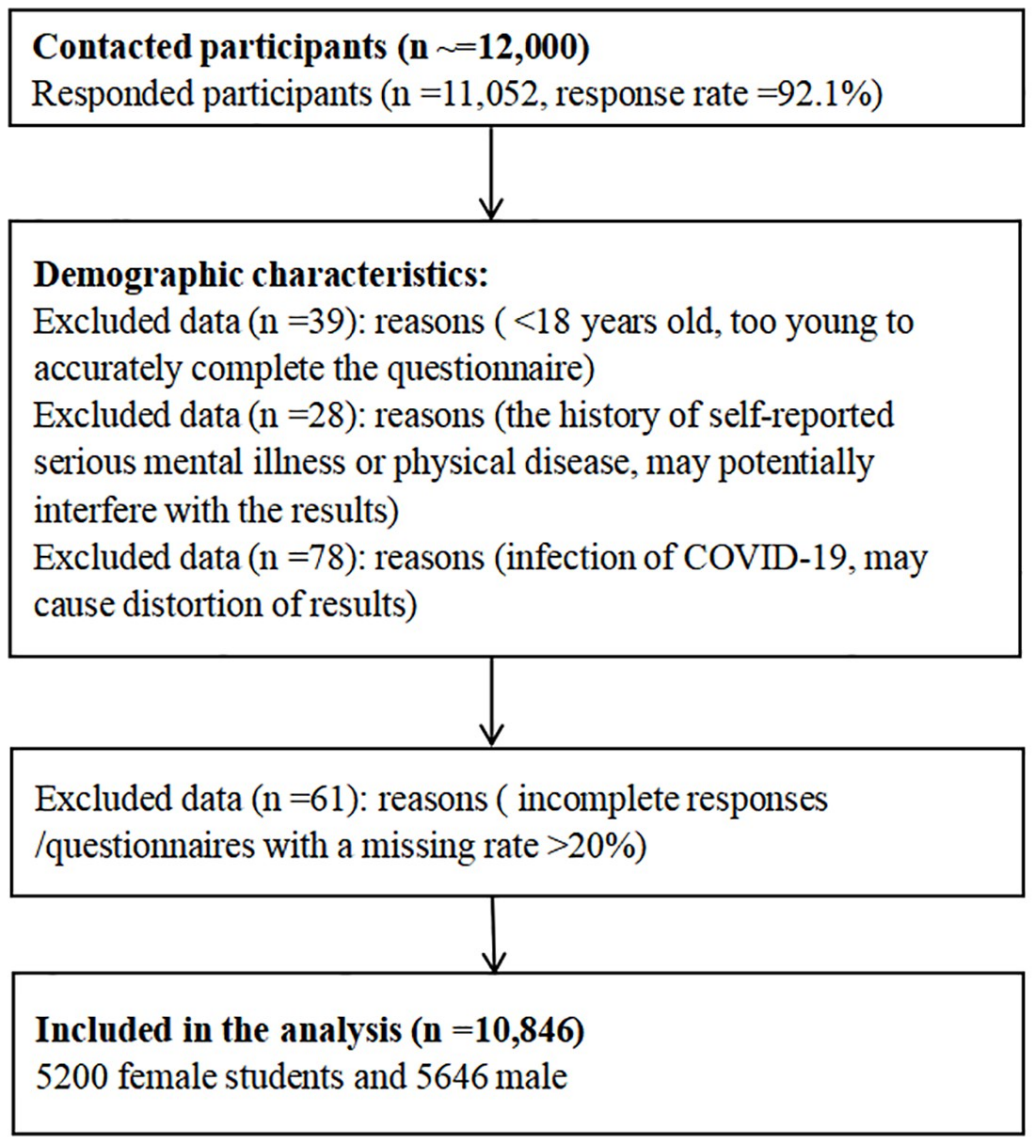
The procedure of participant inclusion or exclusion.

A total of 12,000 Chinese overseas students aged 18 and above, residing in 37 foreign countries across 5 continents and remaining uninfected throughout the pandemic, were recruited for this study. Participants with self-reported histories of severe mental or physical illnesses will be excluded. The selection process is further explicated in Fig 1. After collecting 12,000 responses, a comprehensive analysis was conducted with a refined sample size of 10,846 participants (Fig 1). Additionally, the Research Ethics Committee of Wuhan Sports University granted approval for the study. (Application No: EAN-2021-0807-387). Written informed consent was obtained from each participant, ensuring their voluntary participation in this research. No potentially identifiable human images or data are presented in this study.

## Procedure

In the period spanning from November 2, 2020, to January 31, 2021, a comprehensive multi-country cross-sectional study was undertaken targeting Chinese students studying abroad. The investigation leveraged online survey questionnaires to gather data on mental health and pertinent demographic details, such as gender, age, academic standing, geographical location, institution affiliation, and the duration of experienced anti-epidemic measures. To assemble respondents for the questionnaire, a snowball sampling method was employed, and the survey was disseminated through WeChat, a widely utilized platform among the Chinese overseas student community. Additionally, organizers of Chinese student societies in various foreign educational institutions played a role in circulating the questionnaire via their social media platforms. Prior to participation, we diligently obtained written informed consent from all participating students. The questionnaire administered to the participants aimed to gather information about their mental health experiences during the pandemic. The entire process took approximately 20 minutes.

## Measures

### Demographic information

Participants furnished self-reported demographic details, covering aspects such as gender, region (Asia, North America, Oceania, Europe, Africa), academic progression (undergraduates, postgraduates, PhD students, visiting scholars), and the temporal span of implemented anti-pandemic measures (1–3 months, 4–6 months, 7–9 months, 10–12 months, surpassing 12 months).

### Mental disorder evaluation

For the assessment of mental disorders among Chinese overseas students during the COVID-19 pandemic, a survey was conducted using a 50-item Self-evaluation Table derived from the Symptom Checklist 90 (SCL-90). The scale comprises five dimensions: anxiety, fear, depression, somatization, and stress, each consisting of ten items. Participants rated these items on a five-point Likert scale (1—None, 2—Mild, 3—Moderate, 4—Considerable, 5—Severe) based on the severity of symptom manifestation. Participants gauged the relevance of each mental disorder item to their experiences during the pandemic. A higher total score indicated more severe psychological problems, while a lower score reflected a lower level of mental disorder. Interpretation was as follows: participants with a total score of 90 or above or those scoring higher than 2 in any dimension were considered to have mental disorders. The questionnaire exhibited strong reliability with a Cronbach’s alpha coefficient of 0.90 for the entire instrument, test-retest reliability surpassing 0.71, and internal consistency at 0.86. The alphas for the five dimensions (anxiety, fear, depression, somatization, and stress) were 0.852, 0.864, 0.765, 0.801, and 0.835, respectively.

### Statistical analysis

Descriptive statistics, such as percentages, 95% confidence intervals, means, and standard deviations, were computed to delineate the demographic characteristics. The statistical significance of differences in mental disorders among participants was assessed using the chi-square test. Frequency analyses were conducted to ascertain the prevalence of mental disorders. Multinomial logistic regression analysis was utilized to investigate the relationship between explanatory variables and mental disorders. All data underwent comprehensive analysis using IBM SPSS for Windows (version 26.0, SPSS Inc. Chicago, IL, USA). A two-tailed p-value less than 0.05 was considered statistically significant.

## Results

### Sample characteristics

Table 1 outlines the descriptive characteristics of the study sample. The final analysis comprised 10,846 participants, with 47.9% female students and 52.1% male students. The participants of the study were enrolled in 138 colleges located in 37 countries, with 17.5% in Asia, 20.2% in North America, 18.0% in Oceania, 41.8% in Europe, and 2.5% in Africa. Distribution by academic level included 36.8% undergraduates, 28.5% postgraduates, 25.6% PhD students, and 9.1% visiting scholars. Duration of engagement in anti-pandemic efforts varied, with 19.3% involved for 4–6 months, 31.8% for 7–9 months, 30.1% for 10–12 months, and 8.9% for over 12 months.

**Table 1:** Demographic characteristics of the study population.

Variables	Overall(n = 10,846)		Female(n = 5200)		Male(n = 5646)	
	N(%)	95%CI	N(%)	95%CI	N(%)	95%CI
**Region**						
Asia	1895(17.5%)	(17%, 18%)	887(17.10%)	(16.6%, 17.6%)	1008(17.90%)	(17.2%, 18.6%)
North America	2193(20.2%)	(19.8%, 20.7%)	1052(20.20%)	(19.8%, 20.5%)	1141(20.20%)	(19.9%, 20.5%)
Oceania	1953(18.0%)	(17.8%, 18.2%)	986(20%)	(19.8%, 20.2%)	967(17.10%)	(16.5%, 17.8%)
Europe	4533(41.8%)	(41.5%, 42.2%)	2183(42%)	(41.6%, 42.5%)	2350(41.60%)	(41.2%, 41.9%)
Africa	272(2.5%)	(2.2%, 2.7%)	92(17.70%)	(17.1%, 18.2%)	180(31.90%)	(30.4%, 31.5%)
**Education level**						
Undergraduate	3994(36.8%)	(36.1%, 37.5%)	2213(39.20%)	(38.7%, 39.6%)	1781(34.2%)	(33.6%,34.9%)
Postgraduate	3086(28.5%)	(27.8%, 29.2%)	1434(27.60%)	(27.2%, 27.9%)	1652(29.30%)	(28.9%, 29.8%)
PhD student	2777(25.6%)	(25.1%, 26.1%)	1428(27.50%)	(27.1%, 28%)	1349(23.90%)	(23.4%, 24.5%)
Visiting scholar	989(9.1%)	(8.6%, 9.6%)	557(10.70%)	(10.5%, 10.8%)	432(7.70%)	(7%, 8.4%)
**The duration of anti-pandemic**						
1–3 months	1070(9.8%)	(9.4%, 10.2%)	503(9.7%)	(9.5%, 9.9%)	567(10%)	(9.5%, 10.4%)
4–6 months	2089(19.3%)	(19%, 20.7%)	1160(22.3%)	(21.7%, 23%)	920(16.3%)	(15.8%, 16.9%)
7–9 months	3450(31.8%)	(31.1%, 32.5%)	1805(34.7%)	(34.5%, 34.9%)	1645(29.1%)	(28.4%, 29.9%)
10–12 months	3267(30.1%)	(29.5%, 30.7%)	1510(29.0%)	(28.2%, 29.9%)	1757(31.1%)	(30.8%, 31.5%)
More than 12 months	970(8.9%)	(8.3%, 9.4%)	490(9.4%)	(8.9%, 9.8%)	480(8.5%)	(8.2%, 8.7%)
**Age (years)**						
18–24	4830(44.50%)	(44.2%, 44.7%)	2629(54.4%)	(54.1%, 55.7%)	2201(45.6%)	(45.2%, 46%)
25–29	3402(31.40%)	(30.9%, 31.8%)	1822(53.6%)	(53.2%, 54.1%)	1580(46.4%)	(46.1%, 47.2%)
30–39	1786(16.50%)	(15.9%, 17%)	1105(61.9%)	(61.6%, 62.2%)	681(38.1%)	(37.7%, 38.6%)
40–49	828(7.60%)	(7.2%, 8%)	291(35.1%)	(34.8%, 35.5%)	537(64.9%)	(64.8%, 65%)

Abbreviation: 95%CI: 95% confidence interval.

### Prevalence and correlates of mental disorders

The prevalence of mental disorders among Chinese overseas students is presented in Table 2. Overall, 65.4% of participating students exhibited symptoms of mental disorders. Regional variations were evident, with prevalence rates in Asia, North America, Oceania, Europe, and Africa standing at 70.3%, 56.5%, 61.8%, 69.4%, and 60.9%, respectively, signifying a significant regional disparity (p < 0.001). Furthermore, a noteworthy correlation emerged between the burden of mental disorders and the duration of anti-pandemic measures during the crisis (p < 0.05). However, no statistically significant differences were discerned between male and female students (p > 0.05). Regarding education levels, the distribution of undergraduates, postgraduates, Ph.D. students, and visiting scholars revealed percentages of 66%, 69%, 72.5%, and 41.4%, respectively. Importantly, significant differences were observed among the distinct education groups (p < 0.05). In summary, the findings underscored the significance of regions, education levels, and the duration of anti-pandemic measures as factors associated with mental disorders, while gender exhibited no notable correlations.

**Table 2:** Prevalence of mental disorders among Chinese overseas students.

Variables	Overall (n = 7,090)		*p*
	N (%)	95% CI	
**Region**			
Asia	1332(70.3%)	(70%, 70.7%)	<0.001
North America	1239(56.5%)	(55.9%, 57.2%)	
Oceania	1207(61.8%)	(61.2%, 61.5%)	
Europe	3146(69.4%)	(68.7%, 70.1%)	
Africa	166(60.9%)	(60.3%, 61.4%)	
**Gender**			0.75
Girls	3709(52.3%)	(51.7%, 53.1%)	
Boys	3381(47.7%)	(47.5%, 48%)	
**Education level**			0.212
Undergraduate	2636(66.0%)	(65.2%, 66.7%)	
Postgraduate	2129(69.0%)	(68.5%, 69.4%)	
PhD student	2013(72.5%)	(71.8%, 73.2%)	
Visiting scholar	409(41.4%)	(40.7%, 42.1%)	
**The duration of anti-pandemic**			0.034
1–3 months	844(78.9%)	(78.4%, 79.3%)	
4–6 months	1588(76.0%)	(75.3%, 76.8%)	
7–9 months	2142 (62.1%)	(61.9%, 62.2%)	
10–12 months	1950 (59.7%)	(59.5%, 60%)	
More than 12 months	566(58.4%)	(57.7%, 59.1%)	
**Age (years)**			0.040
18–24 years old	3158.82(65.4%)	(64.7%, 66.1%)	
25–29 years old	2350(69.1%)	(68.8%, 70.5%)	
30–39 years old	1295(72.5%)	(71.9%, 73%)	
40–49 years old	342(41.3%)	(40.8%, 41.9%)	

**Fig 2:**
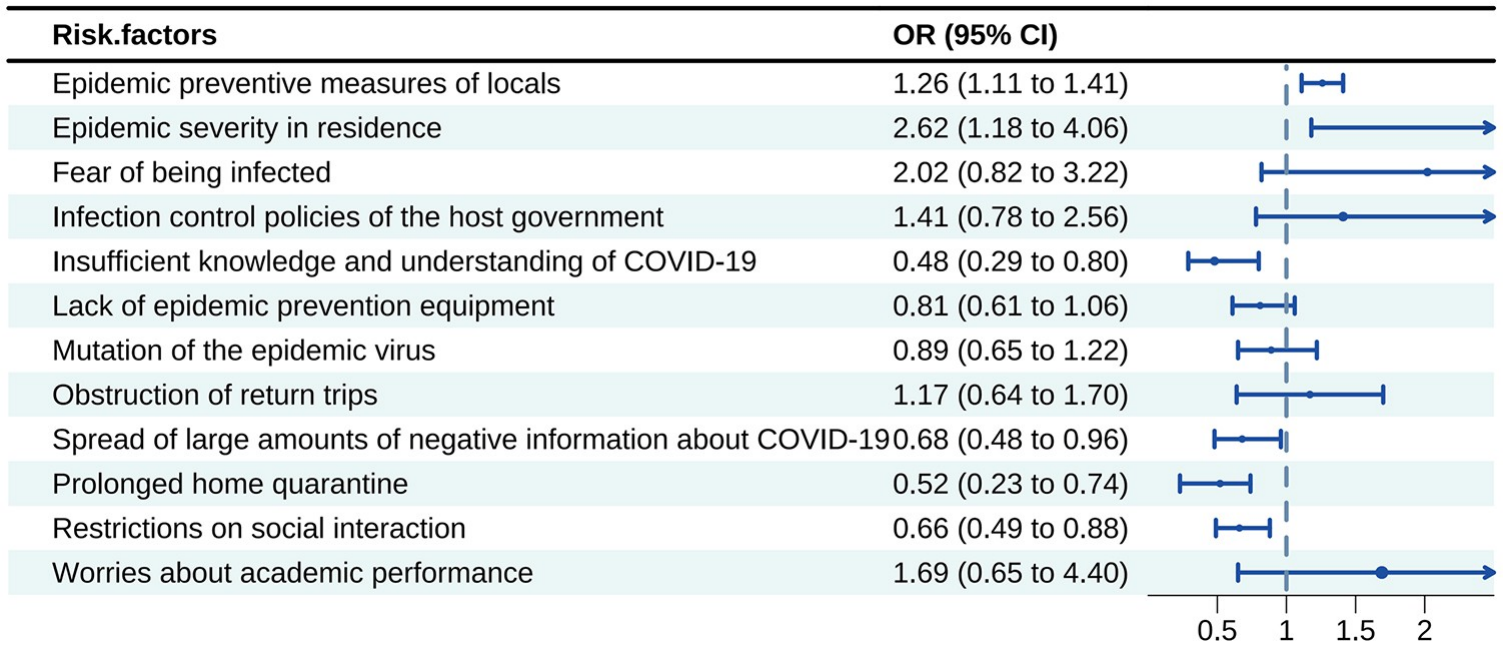
Logistic regression analysis of the risk factors for mental disorders.

Fig 2 presents the findings from the logistic regression analysis. Amidst the COVID-19 pandemic, we identified various risk factors that may contribute to the mental health burden experienced by Chinese overseas students. The key determinants encompassed the seriousness of the epidemic in their residential area, the apprehension of getting infected, anxieties regarding academic performance, the infection control policies implemented by the host government, preventive measures taken locally to counter the epidemic, and challenges encountered in returning to their home country (Fig 2).

## Discussion

The investigation highlights a substantial prevalence of mental disorders among Chinese overseas students during the COVID-19 pandemic. The severity of mental disorders varies based on factors such as region, duration of anti-pandemic measures, education level, and age, while gender differences are not significant. Risk factors contributing to mental disorders include the seriousness of the epidemic in their residential area, the apprehension of getting infected, anxieties regarding academic performance, the host government implemented infection control policies to address the epidemic, and local preventive measures were taken to counter its spread, and challenges encountered in returning to their home country. These findings underscore the importance of tailored interventions and support for Chinese international students in similar circumstances.

A considerable body of evidence indicates that overseas college students tend to experience higher rates of mental disorders compared to the average adult population [8]. Prolonged school closures, class cancellations, shifts to virtual learning, and lifestyle changes during the pandemic have introduced or heightened stressors for Chinese overseas students [9]. The severity of the pandemic itself plays a crucial role in negatively impacting mental health. Mutations of COVID-19 concerns about asymptomatic transmission, and the rising cases, deaths, and reinfections contribute significantly to the psychological distress experienced by Chinese overseas students. Anxiety levels tend to increase when students are personally connected to individuals testing positive for the virus, correlating with elevated depression, anxiety, and aversion [10]. While college life traditionally fosters a sense of belonging for international students [11], the pandemic has disrupted this support system. The disruptions to research projects, academic delays [12], and displacement from on-campus housing have introduced additional stressors for college students [13]. Negative news and updates on the internet fuel anxiety, impacting academic performance and a sense of belonging. Discrimination and isolation in some host countries due to the perception of Chinese overseas students as potential virus carriers [12] further exacerbate their stress, anxiety, and fear [11, 13]. Fearful individuals are more likely to engage in unhealthy behaviors and may experience feelings of hopelessness, creating a detrimental cycle [11, 14]. Furthermore, obstacles to return trips during the pandemic, including closed international air travel and concerns about infection and quarantine, increase the vulnerability of Chinese overseas students to psychopathology. The financial burden of returning to China adds an additional layer of stress [15]. These findings highlight the urgent need for targeted support to address the mental health needs of Chinese overseas students during the pandemic.

Clear regional differences in the mental health status of Chinese overseas students during the COVID-19 pandemic are apparent. Analyses across five continents reveal specific variations in each region. For instance, a meta-analysis indicates that students in Asia report higher levels of depression and anxiety compared to their counterparts in Europe and the Americas [16]. These variations in mental health status among Chinese overseas students during the COVID-19 pandemic may be attributed to differences in cross-cultural and social systems, as well as disparities in the development of the pandemic across different regions [17].

Cultural and normative disparities between Chinese overseas students and locals contribute to divergent views on COVID-19 and preventive measures. The collective nature of Chinese society prioritizes health over leisure [17], evident in the effective control of the epidemic in China. In individualistic countries, striking a balance between pandemic policies and personal freedom is crucial. The uncontrollable nature of the pandemic causes students to be excessively concerned about their health, thereby leading to heightened anxiety among international students [18]. Additionally, the variations in the intensity of physical activity changes among international students across different regions during the pandemic may have significant impacts on their mental health [10].

The stage of pandemic development in host countries also influences the psychological well-being of Chinese overseas students. Varying epidemic prevention environments, encompassing different levels of risk areas and variants of coronavirus, pose greater challenges. Socioeconomic status, degree of positive cooperation from the public, and religious beliefs contribute to variations in preventive and control measures, such as mask-wearing and vaccination [19]. For example, the UK pursued a herd immunity approach, some US colleges required students to vacate on-campus housing, and certain countries faced shortages of epidemic-prevention supplies. If students cannot reconcile to the host government’s situation, their anxiety levels are likely to increase. In summary, when confronted with a threatening situation, the mental well-being of international students can be severely compromised [20], emphasizing the necessity for targeted approaches to provide support.

The duration of anti-pandemic measures does not necessarily correlate with a more serious psychological burden. Over time, negative emotions may gradually diminish as individuals gain competence in dealing with COVID-19 [21], obtain reliable information, adopt preventive measures, and adjust to changed daily life [15, 22]. Mental disorders can be effectively alleviated through the provision of support services by educational institutions, online communities, and families in China. The easing of limitations and reopening of campuses further reduce the psychological burden.

Contrary to previous research, this study finds no significant gender differences in mental disorder problems among Chinese overseas students during the pandemic. Females are considered to be one of the major risk factors associated with mental symptoms, as they exhibit a higher susceptibility to emotional disorders such as generalized anxiety compared to males [23]. Moreover, multiple studies consistently indicate a higher prevalence of mental disorders among women during the pandemic, with female students demonstrating a greater vulnerability to experiencing heightened psychological impact compared to their male counterparts [24, 25]. Possible explanations include the basic consistency of living situations and challenges faced by male and female students studying in the same area. Further research is necessary to explore these inconsistent findings.

Previous research has demonstrated that worries about academic performance positively correlate with psychological problems among Chinese overseas students [5]. Prolonged school closures, class postponements, and the transition to online learning introduce new stressors [24, 25]. The unfamiliarity with online learning methodologies adds to the stress of Chinese overseas students [26]. The reduction or absence of a strong sense of belongingness may explain the connection between COVID-19 and mental disorders.

During the COVID-19 pandemic, it is a new topic to explore whether there are differences in mental disorders among students at different stages of learning. This study has revealed a correlation between educational level and mental disorders. Postgraduate and PhD students faced additional stress in managing research tasks and experimental projects, with unexpected closures and disruptions impeding progress [27, 28]. Delays in graduation and weakened competitiveness in the job market exacerbated their mental health challenges [29, 30]. The primary challenge faced by Chinese visiting scholars abroad lies in the preservation of their scholarly enthusiasm and research continuity. It is crucial for them to effectively address the feelings of isolation and anxiety that may arise due to language barriers, navigate through cultural differences and work-related challenges, adapt to the academic lifestyle abroad, and prioritize personal safety [31].

Simultaneously, the varying levels of education among overseas students indirectly reflect age disparities, which contribute to different psychological disorders experienced during the COVID-19 pandemic. The available evidence indicates that older overseas students, with their extensive life experiences, demonstrate effective stress management skills and focus on the positive aspects of challenges by actively engaging in stress-reducing activities to regulate their emotions [30, 32]. Moreover, young overseas students may demonstrate diminished psychological resilience in adapting to environmental fluctuations and economic pressures [29] and thus are more susceptible to intense reactions when confronted with stressful occurrences such as the pandemic. The conclusion of our research is congruent. The mental disorders associated with the pandemic disproportionately affect different age groups, highlighting the need for tailored interventions and support systems to address the specific challenges faced by each demographic.

## Implications and limitations

The findings have several implications: Given the high prevalence of mental disorders, tailored interventions should be developed to address the specific needs of Chinese overseas students. These interventions can include psychological counselling, mental health awareness programs, and support networks. The study highlights the need for global cooperation in addressing the mental health of international students. Collaborative efforts between home and host countries, educational institutions, and international organizations are essential to create a supportive environment. Governments should play a proactive role in enhancing psychological counselling and humanistic care for key populations, particularly during public health incidents. This can contribute to reducing the risk of mental disorders and promoting overall well-being.

Despite its contributions, this study has certain limitations that should be considered: The use of online platforms for questionnaire dissemination led to uneven and incomplete regional distribution. The diversity of Chinese overseas students on 5 continents may affect the generalizability of the findings. The reliance on self-reported online questionnaires introduces the possibility of recall and reporting bias. While efforts were made to use a scale adapted from the SCL-90 with high reliability and validity, the subjective nature of self-reporting is an inherent limitation. The study failed to collect crucial background information, thereby impeding a comprehensive understanding of the risk factors that influence mental disorders.

## Conclusions

In conclusion, this study sheds light on the mental health challenges faced by Chinese overseas students during the COVID-19 pandemic. The identified correlations between region, education level, duration of anti-pandemic measures, and age with mental disorders emphasize the need for targeted support. Key risk factors, include the seriousness of the epidemic in their residential area, the apprehension of getting infected, anxieties regarding academic performance, the host government implemented infection control policies, and local authorities took preventive measures to counter the spread of the epidemic, and challenges encountered in returning to their home country. Enhancing psychological counselling and humanistic care for key populations is crucial for mitigating the risk of mental disorders among Chinese overseas students during similar public health incidents.
